# Bone Tissue Engineering: Recent Advances and Translation to Clinical Application

**DOI:** 10.3390/jfb17020075

**Published:** 2026-02-05

**Authors:** Fernando P. S. Guastaldi, Bhushan Mahadik

**Affiliations:** 1 Division of Oral and Maxillofacial Surgery, Department of Surgery, Massachusetts General Hospital, Boston, MA 02114, USA; 2 Department of Oral and Maxillofacial Surgery, Harvard School of Dental Medicine, Boston, MA 02115, USA; 3 Prellis Biologics, Berkeley, CA 94710, USA; mahadikb@gmail.com

## 1. Introduction

Large bone defects resulting from trauma, tumor resection, congenital anomalies, infection, or revision surgery represent a persistent and unresolved challenge in orthopedic, maxillofacial, and reconstructive surgery [1,2]. These defects frequently exceed the bone’s intrinsic regenerative capacity and are associated with delayed union, non-union, functional impairment, and long-term morbidity [1,2]. From a global health perspective, the burden is substantial: musculoskeletal conditions are among the leading causes of disability worldwide, and complex bone reconstruction procedures contribute significantly to healthcare costs, prolonged rehabilitation, and reduced quality of life for affected patients [3].

Although bone is a highly dynamic tissue capable of self-repair under physiological conditions, critical-sized defects lack the biological and mechanical environment necessary for spontaneous healing [4]. Current gold-standard clinical approaches—including autografts, allografts, and vascularized bone transfers—remain limited by well-documented drawbacks, including donor-site morbidity, limited graft availability, risk of infection and immune rejection, unpredictable resorption, suboptimal anatomical fit, and high procedural complexity [5]. Despite incremental refinements in surgical technique and fixation strategies, these limitations continue to motivate the search for more effective, scalable, and patient-specific regenerative solutions [1,2].

Over the past two decades, bone tissue engineering (BTE) has emerged as a promising interdisciplinary strategy to address these challenges by combining biomaterial scaffolds, cells, and bioactive signals to recapitulate key aspects of native bone regeneration. Advances in computer-assisted design and three-dimensional (3D) printing technologies have further accelerated this field, enabling the fabrication of patient-specific scaffolds with precisely controlled architecture, porosity, and mechanical properties. These technologies allow implants to be tailored to defect geometry and load-bearing requirements, while also facilitating spatial control over biological cues that guide osteogenesis and vascularization [1,2,3,6,7].

Concurrently, growing evidence highlights the critical role of the host immune response in determining the fate of tissue-engineered constructs [8]. Rather than acting solely as a barrier to implantation, the immune system actively orchestrates bone healing through tightly regulated inflammatory, angiogenic, and remodeling phases. Immunomodulation is now recognized as a central design consideration in BTE, influencing scaffold integration, stem cell behavior, and long-term regenerative success [8]. As a result, contemporary strategies increasingly seek to engineer materials and constructs that interact constructively with the immune microenvironment rather than merely avoiding adverse reactions [8].

Together, these technological and biological advances have repositioned BTE and 3D printing from experimental concepts toward clinically relevant, translational platforms. By offering minimally invasive, personalized, and biologically instructive alternatives to traditional bone grafting, these approaches have the potential to improve functional outcomes, reduce complications, and ultimately transform the management of large bone defects [9,10]. This evolving landscape provides the foundation for the contributions assembled in this Special Issue, which collectively examine both the opportunities and the remaining barriers to the clinical realization of engineered bone regeneration.

## 2. Special Issue Highlights

The Special Issue, entitled “Bone Tissue Engineering: Recent Advances and Translation to Clinical Application,” brings together experimental, preclinical, and perspective-driven contributions that collectively highlight both the promise and the complexity of translating biomaterial-based strategies into effective bone regeneration therapies. The articles span scaffold design, biofunctionalization, stem cell integration, and clinical contexts, offering a coherent snapshot of where the field stands today and where it must evolve to achieve reliable clinical impact.

Guagnini et al. (2025) [11] investigated alginate scaffolds modified with fibronectin and bioactive glass, revealing an instructive divergence between in vitro and in vivo performance. While primary cell proliferation in vitro remained limited, the scaffolds demonstrated effective host integration in vivo, underscoring the limitations of conventional in vitro assays in predicting regenerative outcomes. This work underscores the importance of biological context and host response in evaluating biomaterials and highlights how immunomodulation, vascularization, and tissue–material interactions can outweigh simple metrics of early cell proliferation when assessing translational potential.

Sillmann et al. (2025) [12] focused on scaffold architecture by comparing 3D-printed β-tricalcium phosphate (β-TCP) constructs cultured dynamically with primary bone-marrow-derived stromal cells. Their study demonstrates how architectural parameters and mechanical stimulation synergistically influence cell distribution and osteogenic behavior. By emphasizing the role of dynamic culture conditions, this work advances the field’s understanding of how bioreactor-based approaches can better mimic physiological environments and enhance the predictive value of preclinical scaffold testing.

Santos-Silva et al. (2024) [13] presented a compelling large-animal study combining PLLA/graphene oxide scaffolds with a canine-placenta-derived hydrogel and mesenchymal stem cells for mandibular bone repair in goats. This integrative approach exemplifies the growing trend toward multifunctional constructs that combine structural support, bioactive cues, and viable cells. The use of a clinically relevant defect model enhances the study’s translational relevance and highlights the opportunities and challenges of scaling complex, cell-laden biomaterial systems toward real-world applications.

Guerrero et al. (2024) [14] systematically optimized filament-based TCP scaffold designs to enhance osteoconduction and bone augmentation in in vivo rabbit models. Their work provides valuable insights into how filament spacing, orientation, and overall scaffold geometry influence bone ingrowth and regeneration. By linking design parameters directly to biological outcomes, this study underscores the critical role of rational scaffold engineering and provides practical guidance for the development of bone substitutes for clinical use.

Quek et al. (2024) [15] contribute a comprehensive perspective on stem-cell-based therapies for critical-sized segmental bone defects, critically examining current trends and translational barriers. Their analysis highlights key challenges, including cell sourcing, regulatory complexity, manufacturing scalability, and reproducibility of outcomes. By situating experimental advances within a clinical and regulatory framework, this article serves as a timely reminder that successful translation depends not only on biological efficacy but also on robust development pathways and realistic clinical strategies.

He et al. (2024) [16] addressed regenerative cranioplasty, reviewing biomaterials currently used in clinical practice and discussing emerging alternatives and future challenges. Their work bridges basic biomaterials research and neurosurgical applications, emphasizing mechanical compatibility, infection risk, long-term integration, and patient-specific design. This clinically grounded review underscores the need for interdisciplinary collaboration to advance next-generation cranial implants from concept to routine care.

## 3. Conclusion Remarks

Taken together, the contributions in this Special Issue reflect a clear inflection point in the evolution of BTE, marking its transition from predominantly exploratory, proof-of-concept research to clinically informed, application-driven investigation. Rather than focusing solely on demonstrating feasibility under idealized laboratory conditions, the studies increasingly engage with the biological, mechanical, and logistical complexities that define real-world bone repair. This shift signals a growing recognition that translational success in large-bone regeneration must be judged not only by isolated performance metrics but by the robustness, reproducibility, and clinical relevance of engineered solutions across diverse and demanding scenarios [2,3,7].

Collectively, the works underscore that meaningful progress in bone regeneration cannot be achieved solely through incremental refinements in material chemistry or scaffold geometry. While these elements remain essential, they must be embedded within a broader framework that integrates rational biomaterial design with a deep understanding of host biology, including immune modulation, vascularization, and tissue remodeling. Equally important is the use of biologically relevant and predictive preclinical models, coupled with dynamic, physiologically informed evaluation systems that more accurately recapitulate the in vivo environment and enhance the translational value of experimental findings [2,3,7,8].

Moreover, this Special Issue highlights the need to incorporate clinical, regulatory, and manufacturing considerations early in the development pipeline [3,7]. Issues such as scalability, reproducibility, quality control, cost-effectiveness, and regulatory compliance are no longer peripheral concerns, but central determinants of whether promising technologies can ultimately reach patients [3,7]. By explicitly addressing these dimensions alongside experimental innovation, the contributions presented here help to close the persistent gap between laboratory success and clinical adoption. In doing so, this Special Issue provides a timely and trustworthy resource for researchers, engineers, and clinicians committed to advancing reliable, reproducible, and clinically viable bone regeneration therapies into routine clinical practice.

## Data Availability

No new data were created or analyzed in this study. Data sharing is not applicable to this article.
